# Longstanding Transcriptional Activation of APOA1 and PON1 in Human Hepatocytes by CRISPR/dCas9 Technology: Transcriptomic Profile and Crosstalk with Endothelial Cells

**DOI:** 10.3390/ijms27135951

**Published:** 2026-07-02

**Authors:** Jessica I. C. Haratau, Loredan S. Niculescu, Teodora Barbalata, Gabriela M. Sanda, Elena V. Fuior, Shlomo Sasson, Anca V. Sima, Camelia S. Stancu, Laura Toma

**Affiliations:** 1 Lipidomics Laboratory, Institute of Cellular Biology and Pathology “Nicolae Simionescu” of the Romanian Academy, 8, B.P. Haşdeu Street, 050568 Bucharest, Romania; jessica.ciobanu@icbp.ro (J.I.C.H.); loredan.niculescu@icbp.ro (L.S.N.); teodora.barbalata@icbp.ro (T.B.); gabriela.sanda@icbp.ro (G.M.S.); anca.sima@icbp.ro (A.V.S.); camelia.stancu@icbp.ro (C.S.S.); 2 Gene Regulation and Molecular Therapies Laboratory, Institute of Cellular Biology and Pathology “Nicolae Simionescu” of the Romanian Academy, 8, B.P. Haşdeu Street, 050568 Bucharest, Romania; elena.fuior@icbp.ro; 3 Department of Pharmacology, Institute for Drug Research, Faculty of Medicine, The Hebrew University of Jerusalem, Jerusalem 9112002, Israel; shlomo.sasson@mail.huji.ac.il

**Keywords:** apolipoprotein A1, paraoxonase 1, CRISPR/dCas9, transcriptomic profile, RNAseq, in vitro experimental model, hepatocytes, primary endothelial cells

## Abstract

Apolipoprotein A1 (APOA1) and paraoxonase 1 (PON1) are key proteins of high-density lipoproteins (HDL). The aim of the present study was to obtain and characterize an in vitro model for endogenous APOA1 and PON1 longstanding upregulation in hepatocytes that can be further used to decipher the mechanisms of their protective action. Cultured human hepatocytes (HuH-7 cell line) were transfected with CRISPR/dCas9 activation plasmids targeting *APOA1*/*PON1* genes. Following selection with specific antibiotics, RNA sequencing was used for the transcriptomic characterization of the transfected hepatocytes. The functionality of the secreted APOA1/PON1 was evaluated as the capacity of the conditioned medium (CM) from transfected HuH-7 to modulate the oxidative and inflammatory stress in TNFα-activated primary human umbilical endothelial cells (HUVEC). The results showed that: (1) a robust, longstanding upregulation (46 days) of endogenous *APOA1*/*PON1* was obtained after CRISPR/dCas9 transfection and antibiotics selection; (2) *APOA1*/*PON1* upregulation led to a modified transcriptomic profile and increased the expression of several antioxidant genes in transfected hepatocytes as demonstrated by RNAseq analysis; (3) secreted APOA1/PON1 were functional as demonstrated by the CM ability to reduce the levels of reactive oxygen species and inflammatory markers (VCAM-1, MCP-1) in TNFα-activated HUVEC. In conclusion, we achieved an experimental model of successful longstanding upregulation of endogenous APOA1 and PON1 in human hepatocytes. The targeted proteins are secreted in a functional form and can be used for deciphering their complex mechanism of protective action in various pathological conditions.

## 1. Introduction

Liver is a central metabolic organ, with key roles in the regulation of lipid metabolism, oxidative stress and systemic inflammation [1]. Accumulating evidence indicates a functional crosstalk between the liver and the cardiovascular system. In this framework, it has been shown that a healthy liver contributes to the vascular tree well-being, while its dysfunction is associated with the inception and progression of cardiovascular diseases (CVD), the clinical manifestation of atherosclerosis, which is the main cause of mortality and morbidity worldwide [1].

High-density lipoproteins (HDL) are macromolecular complexes that play an anti-atherosclerotic role. HDL are secreted mainly by hepatocytes, and their beneficial effects are partially mediated by two major proteins: apolipoprotein A1 (APOA1), the main structural protein, and paraoxonase 1 (PON1), an antioxidant enzyme. APOA1, in the lipid-free form or associated with HDL, participates in the reverse cholesterol transport process and has antioxidant and anti-inflammatory properties [2,3]. PON1 exerts the antioxidant protection of HDL and reduces the levels of the inflammatory proteins expressed by the endothelial cells exposed to pro-atherogenic conditions [2,3]. However, the mechanisms of anti-atherosclerotic action of HDL and their associated proteins are not fully understood, more investigations being required [4].

Since its discovery, CRISPR/Cas9 technology offered innovative possibilities for the precise manipulation of cellular DNA. The CRISPR/Cas9 system comprises a guiding RNA (gRNA) which directs Cas9 nuclease to a specific site of the host DNA based on base pair complementarity, thus allowing the gene editing (e.g., knock-ins or knock-outs). Dead Cas9 (dCas9) is a catalytically inactive Cas9, with lost endonucleases activity, which retains the ability to bind specific DNA targets, based on a single gRNA (sgRNA) sequence. These characteristics of dCas9 were exploited in the development of the CRISPR/dCas9 activation system, which contains transcriptional activator complexes in addition to the dCas9 enzyme and the customizable gRNA. The interaction of dCas9/sgRNA and transcriptional effector complexes with gene promoters enables specific, robust transcriptional activation of the target genes [5,6,7,8].

Attempts were made to increase APOA1 or PON1 levels by generating transgenic cells or animals overexpressing human APOA1, often using viral vectors to transfer the foreign DNA encoding these proteins to the host [9,10,11]. In contrast to knock-in systems, CRISPR/dCas9 system is used for the specific transcriptional activation of endogenous genes, without affecting the genome sequence, minimizing the toxicity and offering a more physiological way for cells to produce self-functional proteins. The aim of the present study was to obtain and characterize an experimental model for longstanding up-regulation of APOA1 and PON1 in cultured human hepatocytes using the advantages of the CRISPR/dCas9 activation system. This experimental model could be a novel, physiologically relevant platform to uncover new mechanisms of action of these proteins or to develop a therapeutic approach. Deep transcriptomic analysis using the RNA sequencing approach was employed to characterize the transfected hepatocytes for the metabolic pathways that can be triggered by the upregulation of endogenous APOA1 or PON1. The functionality of the secreted APOA1 and PON1 was evaluated by assessing their capacity to modulate the oxidative and inflammatory stress in tumor necrosis factor α (TNFα)-activated primary human endothelial cells.

## 2. Results

### 2.1. Time-Course Analysis of APOA1 and PON1 Expression in Selected and Non-Selected Transfected HuH-7 Cells

**Figure 1:**
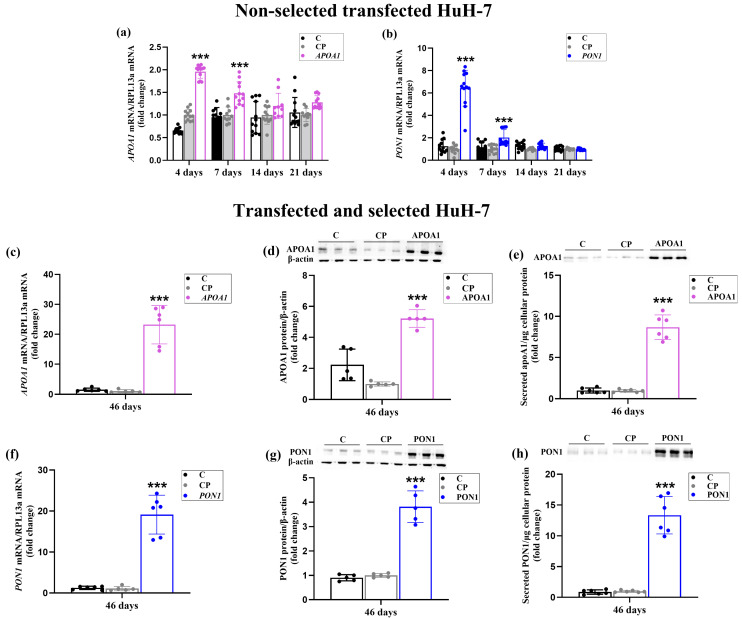
APOA1 and PON1 levels in selected and non-selected transfected HuH-7 cells after CRISPR/dCas9 transfection. *APOA1* and *PON1* mRNA (**a**,**b**) in transfected and non-selected HuH-7 cells. *APOA1* mRNA (**c**), intracellular APOA1 protein (**d**) and secreted APOA1 level (**e**) in HuH-7 cells transfected with plasmids for *APOA1* activation and selected with antibiotic mix, at 46 days after the transfection. *PON1* mRNA (**f**), intracellular PON1 protein (**g**) and secreted PON1 level (**h**) in HuH-7 cells transfected with plasmids for PON1 activation and selected with an antibiotic mix, at 46 days after the transfection. The intracellular protein level is expressed relative to β-actin, and the secreted proteins in the culture medium are normalized to the total cell protein. All data are expressed as fold change versus cells transfected with control plasmids (CP) and presented as mean ± SD. *** *p* < 0.001 versus CP.

After transfection, the gene and protein expression of APOA1 and PON1 were determined in selected and non-selected HuH-7 cells at multiple time-points. The real-time PCR analysis showed that four days after the transfection, *APOA1* mRNA level increased 2-fold (*p* < 0.001) and *PON1* mRNA 6-fold (*p* < 0.001) in comparison with the levels in cells transfected with control plasmid (CP) (Figure 1a,b). However, the gene expression levels of both proteins were gradually reduced in time in the non-selected cells, so that after 14 days a return to the initial CP values was observed (Figure 1a,b). In contrast, the gene expression of *APOA1* and *PON1* in the cells selected with the specific antibiotics remained upregulated even 46 days after transfection, reaching higher values of mRNA compared to the ones at 4 days (approx. 20-fold for both APOA1 and PON1, *p* < 0.001, vs. CP) (Figure 1c,f). Consistently, the protein levels in the cell lysates and the amount of the secreted APOA1 and PON1 in the conditioned media (CM) increased slowly over time (Supplementary Figure S1) and remained highly upregulated at 46 days (5-fold for APOA1 and 4-fold for PON-1 in cell lysate, *p* < 0.001; approx. 10-fold for APOA1 and 13-fold for PON1 in cells’ media, *p* < 0.001) as demonstrated by Western blot analysis (Figure 1d,e,g,h).

### 2.2. Transcriptome Profiling of HuH-7 Cells with Longstanding Upregulated APOA1 or PON1

**Figure 2:**
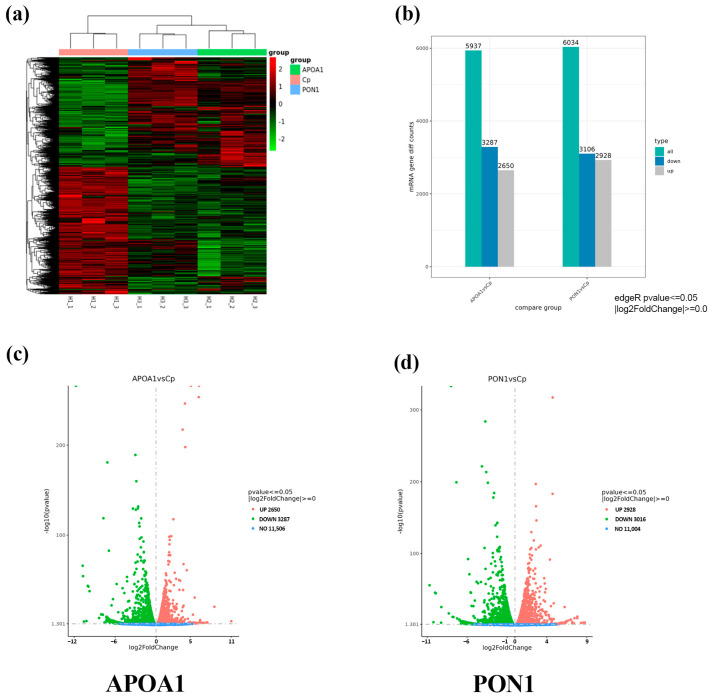
Distribution of differentially expressed (DE) genes identified by RNAseq analysis in transfected and selected hepatocytes for APOA1 or PON1. (**a**) Heatmap diagram with functional group connections for DE genes distribution in individual samples of transfected and selected HuH-7 cells: red—upregulated genes, green—down-regulated genes; (**b**) The number of upregulated (gray) and down-regulated (blue) DE genes identified in HuH-7 cells with transcriptional activated *APOA1* and *PON1*, normalized to HuH-7 cells transfected with control plasmid (CP). The statistical thresholds applied to identify the DE genes were edgeR adj.*p* value ≤ 0.05 and |log2FoldChange| ≥ 0.0; (**c**,**d**) Volcano plots expressing DE gene identified in selected HuH-7 hepatocytes with upregulated *APOA1* (**c**) or upregulated *PON1* (**d**) normalized to HuH-7 cells transfected with control plasmid, (CP). The statistical thresholds applied to identify the DE genes were *p* value ≤ 0.05 and |log2FoldChange| ≥ 0. Red—upregulated genes, green—down-regulated genes, blue—unchanged genes.

To characterize the metabolic changes in the selected HuH-7 cells with endogenously upregulated *APOA1* or *PON1*, we performed bulk-RNAseq analysis of total RNA isolated from these cells. Results showed a significant change in the transcriptomic profile of the selected hepatocytes with transcriptionally activated *APOA1* and *PON1*, reflecting a modified phenotype compared to CP-treated cells, as indicated by the heatmap diagram with functional group connections for differentially expressed (DE) genes (Figure 2a). Furthermore, a significant number of DE genes reached the statistical significance threshold (edgeR adj.*p*-value ≤ 0.05 and |log2FoldChange| ≥ 0). Accordingly, over 5900 DE genes in APOA1 group, and over 6000 DE genes for PON1 cells were recorded. The upregulated and down-regulated DE genes distribution was represented as Volcano plots. These data demonstrate the significantly modified transcriptome profiles for both APOA1- and PON1-transfected and selected hepatocytes (Figure 2b–d).

### 2.3. Enrichment Analysis of Differentially Expressed Genes Associated with Oxidative Signaling Pathways

**Figure 3:**
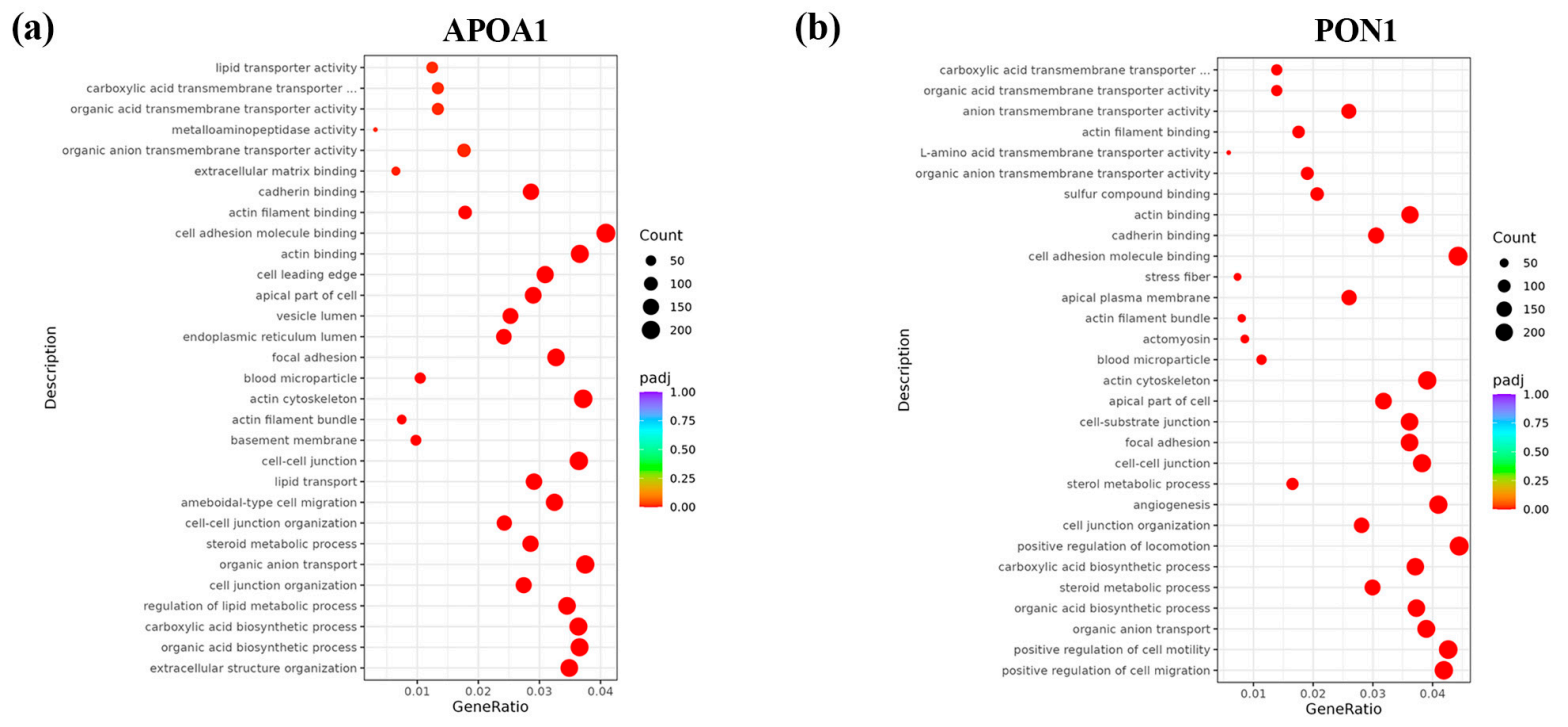
Functional enrichment analysis for DE genes in HuH-7 cells with upregulated APOA1 (**a**) and PON1 (**b**) using DAVID tools and Gene Ontology (GO) database. Dot plots illustrate top 10 biological processes (BP, top), molecular functions (MF, middle) and cellular components (CC, bottom) associated with up- and down-regulated DE genes identified using the cutoff thresholds adj*.p*-value ≤ 0.05 and |log2FoldChange| ≥ 0. Dot size depicts the identified gene count in the functional enrichment group, the dot color represents the adj*.p*-values of predicted functional enrichment, and GeneRate is the ratio of the identified genes in the functional group to total number of the DE genes.

We used the Database for Annotation, Visualization and Integrated Discovery (DAVID) tools v2025_2 and Gene Ontology (GO) database to perform a functional enrichment analysis for biological processes (BP) associated with the identified DE genes (significantly changed, with adj*.p*-value < 0.05) in hepatocytes with transcriptionally activated *APOA1* or *PON1*. The metabolic profiles were significantly different between the two groups of selected hepatocytes (Figure 3). Accordingly, using STRING analysis of DE genes identified in DAVID, we showed that in the APOA1 group most of the BP classes were related to cholesterol metabolism, such as cholesterol metabolic processes (GO:0008203), while many BP related to oxidative stress were found to be altered by transcriptional activation in both groups, such as the response to oxidative stress (GO:003459) (Figure 3).

**Figure 4:**
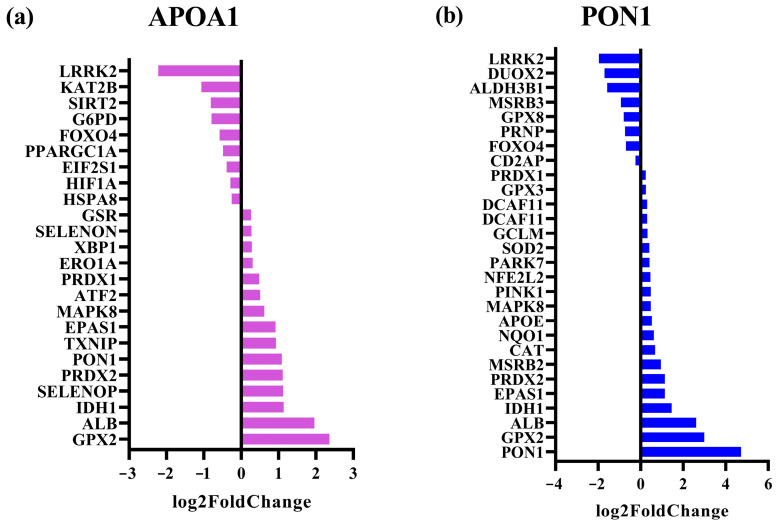
Differentially expressed genes in HuH-7 cells with upregulated APOA1 or PON1. Differentially expressed (DE) genes from GO biological process (BP) showing the response to oxidative stress (GO:0034599) in selected HuH-7 cells with transcriptional activated APOA1 (**a**) or PON1 (**b**).

We also analyzed the overlapping or uniquely DE genes associated with the longstanding transcriptional activation of APOA1 and/or PON1 in hepatocytes and we constructed a Venn diagram (Supplementary Material—Figure S2 and Table S3). We further performed a KEGG functional enrichment analysis using DAVID and STRING platforms for shared DEGs and we observed that the most important modulated pathway is the Metabolic pathway (282 genes, adj.*p*-value = 3.13 × 10^−5^) (Supplementary Material—Figure S3). We further identified the relative expression changes in DE genes identified to belong to the response to oxidative stress (GO:0034599) in hepatocytes with upregulated *APOA1/PON1* compared to CP. We found that the gene that encoded glutathione peroxidase 2 (*GPX2*) had the highest significant increase in cells with transcriptional activated *APOA1* (log2FC = 2.36 adj.*p*-value = 4.03 × 10^−14^) or *PON1* (log2FC = 3.00, adj.*p*-value = 2.68 × 10^−24^) (Figure 4a,b). In addition, the mRNA expression of albumin (*ALB*) was significantly increased in both APOA1- and PON1-transfected hepatocytes (log2FC = 1.96, adj.*p*-value = 1.98 × 10^−87^, and, respectively, log2FC = 2.61, adj.*p*-value = 5.56 × 10^−194^) (Figure 4a,b). Peroxiredoxin 2 (*PRDX2*), an important antioxidant enzyme, exhibited significantly increased expression in selected hepatocytes with upregulated *APOA1* (log2FC = 1.11, adj.*p*-value = 4.95 × 10^−6^) or *PON1* (log2FC = 1.13, adj.*p*-value = 2.13 × 10^−34^) (Figure 4a,b). Very interesting, we found that *PON1* mRNA was upregulated in the APOA1 HuH-7 cells (log2FC = 1.08, adj.*p*-value = 5.93 × 10^−6^) (Figure 4a). Another important antioxidant enzyme, catalase (*CAT*), was found to be significantly increased only in PON1-transfected hepatocytes (log2FC = 0.68, adj.*p*-value = 8.48 × 10^−7^) (Figure 4b). At lower levels, superoxide dismutase 2 (*SOD2*) mRNA or the master transcription factor that regulates the cellular defense against oxidative stress *NFE2L2* (*NRF2*) were also shown to be slightly upregulated in PON1 HuH-7 cells (Figure 4b).

**Figure 5:**
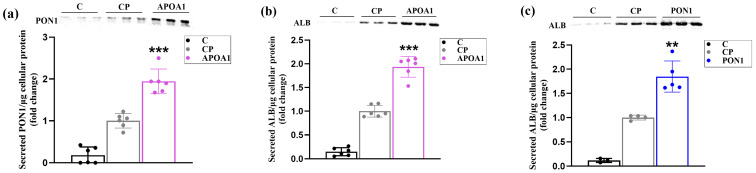
Partial validation of RNAseq results by Western blotting. Secreted PON1 in the conditioned media from APOA1-transfected and selected HuH-7 (**a**); secreted ALB level in the conditioned media from APOA1 (**b**) or PON1 (**c**) transfected and selected HuH-7 cells. Data are expressed as fold change versus cells transfected with control plasmids (CP) and presented as mean ± SD. ** *p* < 0.01, *** *p* < 0.001 versus CP.

We further selected some of DE genes from the metabolic changes, in particular from the oxidative stress-related pathways, for experimental validation of the mechanistic evidence of RNAseq data in transfected HuH-7 cells by measuring their protein expression. To this purpose, the antioxidant PON1 and ALB were analyzed by Western blotting in the CM from transfected and selected HuH-7 cells. Interestingly, the results showed an increase in the protein levels of PON1 in the CM from APOA1 transfected cells. An increase in the ALB levels in the CM from both APOA1- and PON1-transfected HuH-7 cells was also detected (Figure 5a–c).

### 2.4. APOA1- and PON1-Enriched Conditioned Media from Transfected HuH-7 Cells Alleviate TNFα-Induced Oxidative and Inflammatory Stress in Human Primary Endothelial Cells

**Figure 6:**
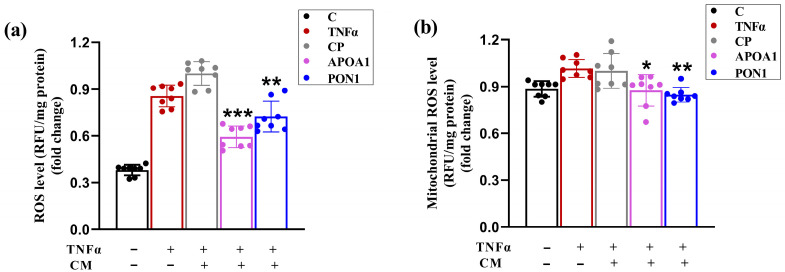
Oxidative stress markers in TNFα-activated HUVECs exposed to conditioned media from HuH-7 cells transfected with APOA1 or PON1 plasmids. ROS levels expressed relative to cellular protein (**a**) and mitochondrial ROS levels expressed relative to cellular protein (**b**) in TNFα-activated HUVECs. Data are presented as mean ± SD. * *p* < 0.05, ** *p* < 0.01, *** *p* < 0.001 versus CP.

The functionality of the secreted APOA1 and PON1 was evaluated as the capacity of the CM from transfected hepatocytes to diminish the oxidative or inflammatory stress in TNFα-activated HUVECs. For that purpose, HUVECs were activated by exposure to TNFα (6 h), and further exposed to CM (for 18 h) from hepatocytes with transcriptional activated *APOA1/PON1*. To validate the secreted APOA1/PON1 functional potential, total reactive oxygen species (ROS) levels, mitochondrial ROS and the expression of vascular cell adhesion molecule 1 (VCAM-1) and monocyte chemoattractant protein 1 (MCP-1) were measured. The results showed that CM from HuH-7 cells with upregulated APOA1 or PON1 reduced TNFα-induced intracellular ROS levels (*p* < 0.001 for APOA1 and *p* < 0.01 for PON1) compared to CM from CP cells, as indicated in Figure 6a. In good agreement, both APOA1- and PON1-enriched CM reduced mitochondrial ROS levels (Figure 6b).

**Figure 7:**
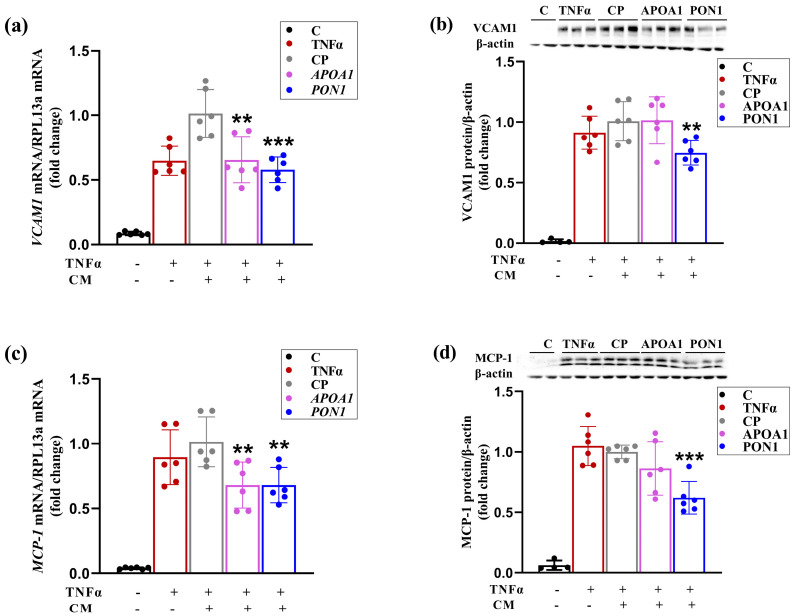
Inflammatory stress markers in TNFα-activated HUVECs exposed to conditioned media from HuH-7 cells transfected with APOA1 or PON1 plasmids. VCAM-1 mRNA (**a**) and protein (**b**), and MCP-1 mRNA (**c**) and protein (**d**) in TNFα-activated HUVECs exposed to CM from transfected HuH-7. The protein level is expressed relative to β-actin. All data are expressed as fold change versus cells transfected with control plasmids (CP) and presented as mean ± SD. ** *p* < 0.01, *** *p* < 0.001 versus CP.

The gene expression of *VCAM-1* and *MCP-1* was significantly decreased by both APOA1- and PON1-CM from transfected HuH-7 (Figure 7a,c). CM from PON1-transactivated HuH-7 significantly decreased the protein expression for VCAM-1 and MCP-1, while APOA1-CM did not reach the statistically significance although a slight decrease is also observed (Figure 7b,d).

## 3. Discussion

Studies on the athero-protective effects of HDL and its main proteins, APOA1 and PON1, are abundant, but new molecular mechanisms concerning their mode of action are needed. The novelty of the present study consists in the use of CRISPR/dCas9 system to upregulate the endogenous APOA1 and PON1 in HuH-7, offering a more physiological perspective to analyze the mechanisms of action of the target proteins. The key findings of the present study are: (1) the CRISPR/dCas9 system was successfully used for the up-regulation of endogenous APOA1 and PON1 gene expression and increase in the secreted proteins; (2) a cellular population with sustained and longstanding upregulated APOA1 and PON1 expression was obtained after the selection of the transfected hepatocytes with specific antibiotics; (3) APOA1 and PON1 longstanding upregulation modified the transcriptomic profiles of the hepatocytes and increased some protective genes, such as, *GPX2*, *ALB*, *PON1*, *CAT*, *SOD2*, *NRF2*, *PRDX2*; and (4) APOA1 and PON1 secreted by the transfected hepatocytes are functional, as indicated by the capacity of APOA1- or PON1-enriched CM to decrease the oxidative or inflammatory stress in TNFα-activated HUVECs.

CRISPR/dCas9 is a system that does not alter the genome sequence, but allows specific regulation of endogenous genes [12]. This characteristic of CRISPR/dCas9 allows the generation of functional proteins with high specificity, with less off-target effects (as compared to CRISPR/Cas9), and without cellular exhaustion. In addition, the simple design and low cost of sgRNA, as well as the easy use in different cell types (including HuH-7) make CRISPR/Cas9 systems a good alternative for gene editing in different pathologies [7,8,13,14,15,16].

The APOA1 and PON1 are two major structural and functional components of HDL. The rationale for including these two proteins in the study is based on their mutual influence on the proper functioning of HDL. Thus, APOA1 is protected against oxidation by PON1, and the latter cannot attach to HDL particles in the absence of APOA1 [17,18]. We report here a successful transcriptional activation of endogenous *APOA1* and *PON1* genes in HuH-7 cells by using the CRISPR/dCas9 system in agreement with previously reported data on Caco-2 cells [19]. In addition, we show here that in the absence of selection antibiotics, the gene expression of *APOA1* and *PON1* slowly decreased, probably due to the higher rate of cellular division of the un-transfected cells over the transfected ones. Thus, the selection with antibiotics of the transfected hepatocytes allowed the generation of a uniform population of cells with transcriptional activated *APOA1* and *PON1* genes, which remained upregulated even 46 days after the transfection.

To characterize these cells, we used the bulk RNAseq profiling and obtained a deep transcriptomic profile of the metabolic changes. Results show that both APOA1 and PON1 transcriptional activation generates hepatocytes with a plasmid-specific phenotype as shown by the GO functional enrichment analysis of transcriptome data. Response to oxidative stress (GO:0034599) is a GO biological processes, which is altered both in ApoA1- and also PON1-transfected hepatocytes. There are only a few relevant data published related to this process [20,21,22,23]. In good agreement with our results, it was shown that administration of APOA1 mimetic peptides is associated, in a dose-dependent manner, with an increased activity of GSH peroxidases in a mouse model of Parkinson disease [20]. Similarly, administration of flaxseed oil diet or beta-sitosterol determined the upregulation of PON1 together with increased CAT, SOD, or GPX activity and CAT expression in the liver or plasma of diabetic [21] or gamma-irradiated rat models [22]. Our results confirm these data which show an association between the levels of *APOA1* or *PON1* with other antioxidant proteins, and in addition, bring new evidence of an interconnection at the transcriptional level between the above-mentioned genes. We report here the upregulation of PON1 in the CM from APOA1-transfected HuH-7 cells, which confirms the results obtained in Caco-2 enterocytes [19], and the increase in secreted ALB, validating the RNAseq results. To the best of our knowledge, we report here for the first time that APOA1/PON1 upregulation increases *GPX2*, *ALB* and *PRDX2* mRNA in hepatocytes. This direct association indicated by RNAseq opens new directions for the identification of new protective mechanisms of APOA1 and PON1.

Data from literature show that APOA1 mimetics can preserve the function of endothelial cells by reducing the oxidative stress [24,25]. PON1 recombinant was also demonstrated to reduce the expression of inflammatory proteins in endothelial cells [26]. In agreement with these studies, we show here that CM enriched in APOA1 and PON1 from hepatocytes decrease the oxidative and inflammatory stress in HUVECs. These results are in good agreement with another study in Caco-2 enterocytes [19] and demonstrate that APOA1 and PON1 are functional. In addition to APOAI and PON1, we also detected a 2-fold increase in ALB in the CM from APOA1/PON1-transactivated HuH-7, and a similar upregulation of PON1 in the CM from APOA1-transactivated HuH-7. However, given the significant increase in the target upregulated proteins (10–13 fold) in CM, we assume that most of the beneficial effects are due to the secreted APOA1 and PON1.

Although promising, the present experimental model has its limitations. One is the fact that HuH-7 cells are a tumoral cell line. The rationale for using this cell line was that the time frame for our experiments was rather long (46 days), requiring long-term maintenance of the cells in culture, which is difficult to obtain with primary cells. HuH-7 have previously been demonstrated to be a good model for CRISPR/Cas9 gene editing in various pathologic conditions [13,14,15,16] or as models to study cellular processes [27], and were successfully used as experimental models to study the metabolism of high-density lipoproteins [28,29]. Although presenting a phenotype consistent with the hepatocytic origin [30], differences between HuH-7 and primary cells or other cell lines are known [31,32]. As expected, it was shown that compared to primary hepatocytes, HuH-7 present an upregulation of genes related to cell cycle control, such as “G1 to S cell cycle control” and “Cell cycle” [31]. In addition, studies of comparative transcriptomic analysis which evaluated three different cell lines (HuH7, HepG2 or HepB3) demonstrated heterogeneity in the expression of genes that regulate oxidative phosphorylation, cholesterol metabolism or DNA damage [31,32]. These processes were not the focus of the present study. However, care needs to be taken when generalizing experimental data, especially for the above-mentioned cellular mechanisms. Moreover, in vitro models do not have the complexity of an in vivo model, in which organs are interconnected so that cellular responses from hepatocytes can be influenced by the crosstalk with other cell types. In addition, we must keep in mind that although present in a free form in vivo, APOA1 and PON1 are mainly associated with HDL. This packaging allows interaction with receptors, such as SR-B1 on the surface of endothelial cells, known to mediate, at least in part, the anti-atherosclerotic effects of HDL [33,34].

## 4. Materials and Methods

### 4.1. Chemicals

RPMI-1640 medium (RPMI), Dulbecco’s Modified Eagle’s Medium (DMEM), penicillin, streptomycin, neomycin, 2′,7′-Dichlorofluorescein diacetate (DCFH-DA), 2′,7′-Bis-(2-Carboxyethyl)-5-(and-6)-Carboxyfluorescein, Acetoxymethyl Ester (BCECF-AM), protease inhibitor cocktail, sodium fluoride, sodium orthovanadate, and bicinchoninic acid solution (BCA) were from Sigma-Aldrich Co. (St. Louis, MO, USA). APOA1 (sc-400499-ACT), PON1 (sc-402701-ACT), and CP (sc-437275) CRISPR/Cas9 activation plasmids, transfection medium (sc-108062) and UltraCruz Transfection Reagent (sc-395739), along with selection antibiotics Hygromycin B solution (SC-29067), Blasticidin S HCl solution (SC-495389), and Puromycin dihydrochloride (SC-108071) were supplied by Santa Cruz Biotechnology (Dallas, TX, USA). Vascular Cell Basal Medium (PCS-100-030) and Endothelial Cell Growth Kit-VEGF (PCS-100-041) were from ATCC (ATCC, Manassas, VA, USA). TNFα (210-TA-020/CF) was from R&D Systems. Fetal bovine serum (FBS) was from GIBCO (ThermoFisher Scientific, Waltham, MA, USA). High-Capacity cDNA Reverse Transcription kit and SyBr Select Master Mix were from Applied Biosystems (Foster City, CA, USA). ECL chemiluminescent substrate was supplied by AppliChem GmbH (Darmstadt, Germany).

### 4.2. Cells and Culture Conditions

Hepatocytes from human hepatocarcinoma (HuH-7 cell line, Cell Lines Service GmbH, Eppelheim, Germany) were cultured in RPMI-1640 supplemented with FBS (10%, *v*/*v*), penicillin (100 U/mL), and streptomycin (0.1 mg/mL). HuH-7 cells at passage 46 were transfected. STR analysis was used to check the genetic variations over passages in the employed HuH-7 cells. A STR match of 94.5–98.2% for HuH-7 was obtained, meaning that the cells are compatible with the original hepatocytes.

Primary human umbilical vein endothelial cells (HUVEC; PCS-100-010, ATCC, Manassas, VA, USA) were grown in Vascular Cell Basal Medium enriched with Endothelial Cell Growth Kit-VEGF according to manufacturer’s instructions. HUVEC at passage 3 were used for the experiment.

### 4.3. Transfection of HuH-7 Cells to Activate the Transcription of APOA1 and/or PON1 Genes Using CRISPR/dCas9 System

HuH-7 cells were seeded into 12 well plates at a density of 70.000 cells/well in complete RPMI media, without antibiotics. At 70–80% confluency, the hepatocytes were transfected using the CRISPR/dCas9 activation plasmids for APOA1, PON1, or CP containing resistance genes for blasticidin, hygromycin B and puromycin. One µg DNA plasmid/mL and 2.5 µL/mL transfection reagent for each transfection condition were used according to the manufacturer’s instructions. After 48 h, the media was changed, and the cells were left to recover for another 48 h.

### 4.4. Selection of Transfected HuH-7 to Obtain Longstanding Upregulation of APOA1 and/or PON1

After transfection, the culture medium was replaced with RPMI supplemented with 10% FBS, containing hygromycin B (50 µg/mL), blasticidin S HCl (2 µg/mL), and puromycin dihydrochloride (1 µg/mL). The culture medium containing selection antibiotics was changed every two days for a period of 12 days to induce the death of the un-transfected cells. After selection, the culture medium was replaced with antibiotics-free RPMI and the transfected and selected HuH-7 were allowed to grow until confluency in complete culture media.

### 4.5. Bulk RNA-Seq Analysis of APOA1 and PON1-Transfected Hepatocytes

Total RNA was extracted from cells using Trizol (Invitrogen, Carlsbad, CA, USA) based on manufacturer’s instructions. An indicator for RNA quality was given by the A260/280 nm ratio, quantified with the Nanodrop Spectrofluorometer (ThermoFisher Scientific). The A260/280 values for the RNA samples were between 1.8 and 1.9, which is accepted as good quality RNA.

Bulk long-RNA sequencing (longRNA-seq) and standard data analysis were done by an external service (Novogene, Cambridge, UK) on total RNA isolated from hepatocytes. The analysis included an additional RNA sample quality control, directional library preparation (rRNA removal), and long-RNA sequencing on NovaSeq X Plus Series (PE150, 12 G raw data per sample). The RNA integrity was verified in a mandatory QC-step using Agilent bioanalyzer performed by Novogene as a standard preparatory step in NGS RNAseq protocol. RNA Integrity Number, RIN, was evaluated and the samples were considered of sufficient quality. Raw data analysis of transcriptomic data from Novogene included data quality control, filtering, mapping to reference genome, correlation analysis, and differential expression analysis.

Bioinformatic analysis of DE genes for functional enrichment analysis was performed using DAVID v2025_2 or GO biological process (BP) database [35,36]. The analyzed DE genes were defined as those with positive log2fold change and adj.*p-*value < 0.05 in each dataset of group samples (APOA1 and PON1), upregulated or down-regulated relative to cells transfected with CP. BP level was defined as the depth of node in AmiGO2 inferred tree view (the lower level, the more general). The depth of biological process was set to 0 and those terms deeper than 3 were considered relevant. Child annotation terms in the same branch were discarded to avoid redundancy. BP terms with adj.*p-*value < 0.05 after applying Benjamini–Hochberg correction and fold enrichment > 2 were accounted for significance. Further analysis for functional gene clusters identified at BP level was performed in each APOA1 and PON1 sample group of DEG dataset (vs. CP) using STRING platform [37], using high confidence interaction score (0.7) and k-means clustering (for a defined number of at least 3 clusters based on their centroids). Specific genes from the functional clusters were further identified in DEG list and histograms created to show log2FoldChange upregulation or downregulation in APOA1 and PON1 groups compared to CP group.

### 4.6. Preparation of Conditioned Media from Selected HuH-7 with Transcriptional Activated APOA1 and PON1

Selected HuH-7 cells at 100% confluency were incubated for 24 h with RPMI without FBS, to reduce potential FBS interference and to obtain the CM enriched in secreted APOA1 or PON1. After collection, the CM were processed by centrifugation at 300× *g* for 5 min, followed by a second centrifugation at 2000× *g* for 25 min to ensure pelleting of any remaining detached cells or cellular debris. The resulting CM were then either stored at −80 °C for subsequent analyses or used to assess their effects on HUVEC function.

### 4.7. TNFα Activation of HUVECs and Incubation with CM from HuH-7 Cells

**Scheme 1:**
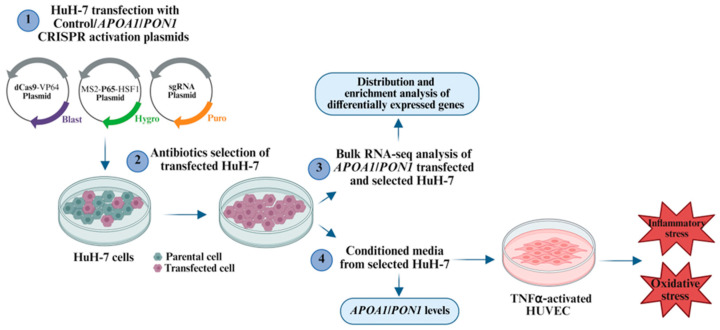
Graphical representation of the experimental design. Created in BioRender. Licensed to Niculescu, L. S. (2026) https://BioRender.com/6nxlu8o.

Confluent HUVECs were stimulated with 10 ng/mL TNFα for 6 h, in the absence of FCS. Following activation, the TNFα-containing culture medium was discarded and CM from transfected HuH-7 cells was further used at a 1:1 ratio with fresh HUVECs culture media. After 18 h incubation, the HUVECs were processed for ROS and, quantitative real-time PCR and Western blot analysis. The experimental design is presented in Scheme 1.

### 4.8. Determination of Total Intracellular ROS

Total intracellular ROS was monitored in HUVECs through the reaction with the oxidant sensitive fluorogenic probe DCFH-DA as in [38]. The fluorescence of DCF in cellular suspension was measured at 435/535 nm using Tecan Infinite M200 and ROS values were normalized to cellular protein and presented as RFU/mg protein.

### 4.9. Measurement of Mitochondrial ROS Levels

Mitochondrial ROS levels were measured by using MitoSOX^TM^ Red Mitochondrial Superoxide Indicator from ThermoFisher Scientific (Carlsbad, CA, USA) according to manufacturer’s instructions. The fluorescence of MitoSOX was measured using Tecan Infinite M200 and the obtained results were normalized to total cellular protein and presented as RFU/mg protein.

### 4.10. Quantitative Real-Time PCR Analysis of Gene Expression

Two μg of total RNA isolated with TRIzol reagent were reverse-transcribed to cDNA using the High-Capacity cDNA Reverse Transcription Kit with MultiScribe Reverse Transcriptase (Applied Biosystems, Waltham, MA, USA), following the recommended protocol. Quantitative real-time PCR was performed using a ViiA7 real-time PCR system (Applied Biosystems). Gene-specific primers were used for human *APOA1* and *PON1* in hepatocytes, and for *VCAM-1*, *MCP-1* in HUVECs; *RPL13A* was used as the housekeeping gene (Table S1, Supplementary Material). Amplification was done using SyBr Select Master Mix (Applied Biosystems). Relative gene expression levels were determined using the “Fit Point Method” and presented relative to CP cells considered 1.

### 4.11. Quantitative Western Blot Analysis of Protein Expression

HuH-7 cells and HUVEC were lysed in RadioImmuno Precipitation Assay (RIPA) buffer supplemented with protease and phosphatase inhibitors, on ice, using Hielscher Ultrasound Processor UP200S (Thermo Fisher Scientific). The total protein concentration was assessed using the Pierce BCA Protein Assay Kit (Thermo Fisher Scientific). To concentrate CM from HuH-7, equal volumes (100 µL) from Control, CP, APOA1 and PON1 cells were precipitated using trichloracetic acid method. Western blot analysis for culture media and lysates samples was performed using standard protocol, that included SDS-PAGE migration, transfer to nitrocellulose membrane and exposure to primary antibodies for APOA1, PON1, ALB (for hepatocytes), VCAM-1, and MCP-1 for HUVECs (Table S2, Supplementary Material). The reference protein for cellular lysates was β-actin and the ratio of target protein to β-actin expression was determined by densitometric analysis of digital images obtained with an Amersham^TM^ ImageQuant^TM^ 800 analyzer and ImageQuant TL version 11 analysis software (Cytiva LifeSciences, Marlborough, MA, USA). Secreted APOA1/PON1 levels in the CM were normalized to total cellular protein. Data for relative protein expression were presented relative to CP cells considered 1.

### 4.12. Statistical Analysis

Statistical analysis and graphical representations were performed using GraphPad Prism 10 software (San Diego, CA, USA). Comparisons between CP versus APOA1- or PON1-transfected HuH-7, and CM-treated HUVECs were made using the Independent Student’s *T*-test. *p* values less than 0.05 were considered statistically significant. The presented data were expressed as mean ± standard deviation (SD) from at least two experiments in triplicate.

## 5. Conclusions

Our data show that longstanding upregulation of *APOA1/PON1* genes can be achieved in HuH-7 cells by using CRISPR/dCas9 technology, followed by selection with specific antibiotics. Bulk RNAseq analysis of transfected hepatocytes indicates a modified transcriptomic profile, demonstrating a direct association between the *APOA1/PON1* upregulation and the increase in genes that are critical for cellular antioxidant protection, such as *GPX2, ALB, PON1, CAT, NRF2, SOD2, PRDX2*. Secreted APOA1/PON1 proteins from transfected and selected HuH-7 cells exert their antioxidant and anti-inflammatory action in TNFα-activated HUVECs. Thus, the present experimental model can be used to confirm, complete and to bring new data regarding the mechanisms of action of APOA1 and PON1 in pathological conditions, and to design future innovative anti-atherosclerotic treatment.

## Data Availability

The raw data supporting the conclusions of this article will be made available by the corresponding authors on request. Raw and processed RNAseq data presented in this study are available on demand and publicly available in ArrayExpress with the accession number E-MTAB-17152.

## Supplementary Materials

The following supporting information can be downloaded at: https://www.mdpi.com/article/10.3390/ijms27135951/s1.

## Abbreviations

The following abbreviations are used in this manuscript:

APOA1	Apolipoprotein A1
PON1	Paraoxonase 1
CM	Conditioned media
HUVEC	Human umbilical vascular endothelial cells
HDL	High-density lipoproteins
CVD	Cardiovascular disease
dCas9	Dead Cas9
sRNA	Single guide RNA
TNFα	Tumor necrosis factor α
CP	Control plasmid
DE	Differentially expressed
DAVID	Database for Annotation, Visualization and Integrated Discovery
GO	Gene Ontology
BP	Biological Processes
GPX2	Glutathione peroxidase 2
ALB	Albumin
PRDX2	Peroxiredoxin 2
CAT	Catalase
SOD2	Superoxide dismutase 2
NRF2	Nuclear Factor, Erythroid 2 Like 2
ROS	Reactive oxygen species
VCAM-1	Vascular cell adhesion molecule 1
MCP-1	Monocyte chemoattractant protein 1
