# Supplementary material for: Longstanding Transcriptional Activation of APOA1 and PON1 in Human Hepatocytes by CRISPR/dCas9 Technology: Transcriptomic Profile and Crosstalk with Endothelial Cells
Source: Int J Mol Sci. 2026 Jul 2;27(13):5951. doi: 10.3390/ijms27135951 (PMC13361997; doi:10.3390/ijms27135951)
Supplement: Supplementary file 1 [file ijms-27-05951-s001.zip › Revised Supplementary file S1.pdf]

Manuscript ID: ijms-4395820

Revised Supplementary Material

Longstanding transcriptional activation of APOA1 and PON1 in human hepatocytes by CRISPR/dCas9 technology – transcriptomic profile and crosstalk with endothelial cells

Jessica I. C. Hărătău[1], Loredan S. Niculescu[1], Teodora Barbălată[1], Gabriela M. Sanda[1], Elena V. Fuior[2], Shlomo Sasson[3], Anca V. Sima[1], Camelia S. Stancu[1], Laura Toma[1,]*

[1]Lipidomics Laboratory, Institute of Cellular Biology and Pathology "Nicolae Simionescu" of the Romanian Academy, 8, B.P. Haşdeu Street, 050568 Bucharest, Romania

[2] Gene Regulation and Molecular Therapies Laboratory, Institute of Cellular Biology and Pathology "Nicolae Simionescu" of the Romanian Academy, 8, B.P. Haşdeu Street, 050568 Bucharest, Romania

[3]Department of Pharmacology, Institute for Drug Research, Faculty of Medicine, The Hebrew University of Jerusalem, 9112002 Jerusalem, Israel.

*Corresponding author:

Laura Toma, PhD

Institute of Cellular Biology and Pathology "Nicolae Simionescu"

8, B.P. Haşdeu Street, 050568 Bucharest, Romania

Phone: +4021 319 4518 / Fax: +4021 319 4519 / E-mail: laura.toma@icbp.ro

Materials and Methods

Table S1. Sequences of human PCR primers

Gene	GeneBank accession number	Sequences of oligonucleotide primers
**APOA-I**	NM_000039.2	FW: 5'-CCCTGGGATCGAGTGAAGGA-3'<br>RV: 5'-CTGGGACACATAGTCTCTGCC-3'
**PON1**	NM_000446.5	FW: 5'-CTATGACTCAGAGAATCCTCCTGCATCAG-3'<br>RV: 5'-CATGGGTGCAAATCGGTCTGTTAGAGC-3'
**VCAM-1**	NM_001078.4	FW: 5'-GATTCTGTGCCCACAGTAAGGC-3'<br>RV: 5'-TGGTCACAGAGCCACCTTCTTG-3'
**MCP1**	NM_002982.4	FW: 5'-GAT CTC AGT GCA GAG GCT CG-3'<br>RV: 5'-TGC TTG TCC AGG TGG TCC AT-3'
**RPL13a**	NM_001101.3	FW: 5'-GTCTTCCCCTCCATCGT-3'<br>RV: 5'-CGTCGCCCACATAGGAAT-3'

Table S2. Specific antibodies used for Western Blot

Antibody	Specificity	Catalogue #	Dilution	Source
Mouse mAb	Human apoA1	sc-69755	1:600	Santa Cruz
Mouse mAb	Human PON1	ab24261	1:500	Abcam
Mouse mAb	Human VCAM-1	sc-13160	1:400	Santa Cruz
Rabbit pAb	Human MCP-1	ab9669	1:1000	Abcam

Mouse mAb	Human albumin	sc-271605	1:1000	Santa Cruz
Mouse mAb	Human β-actin	sc-47778	1:4000	Santa Cruz
	Rabbit Anti-Mouse IgG H&L (HRP)	ab6728	1:10000	Abcam
	Rabbit Anti-Goat IgG H&L (HRP)	ab6741	1:10000	Abcam
	Goat Anti-Rabbit IgG H&L (HRP)	ab6721	1:10000	Abcam

Results

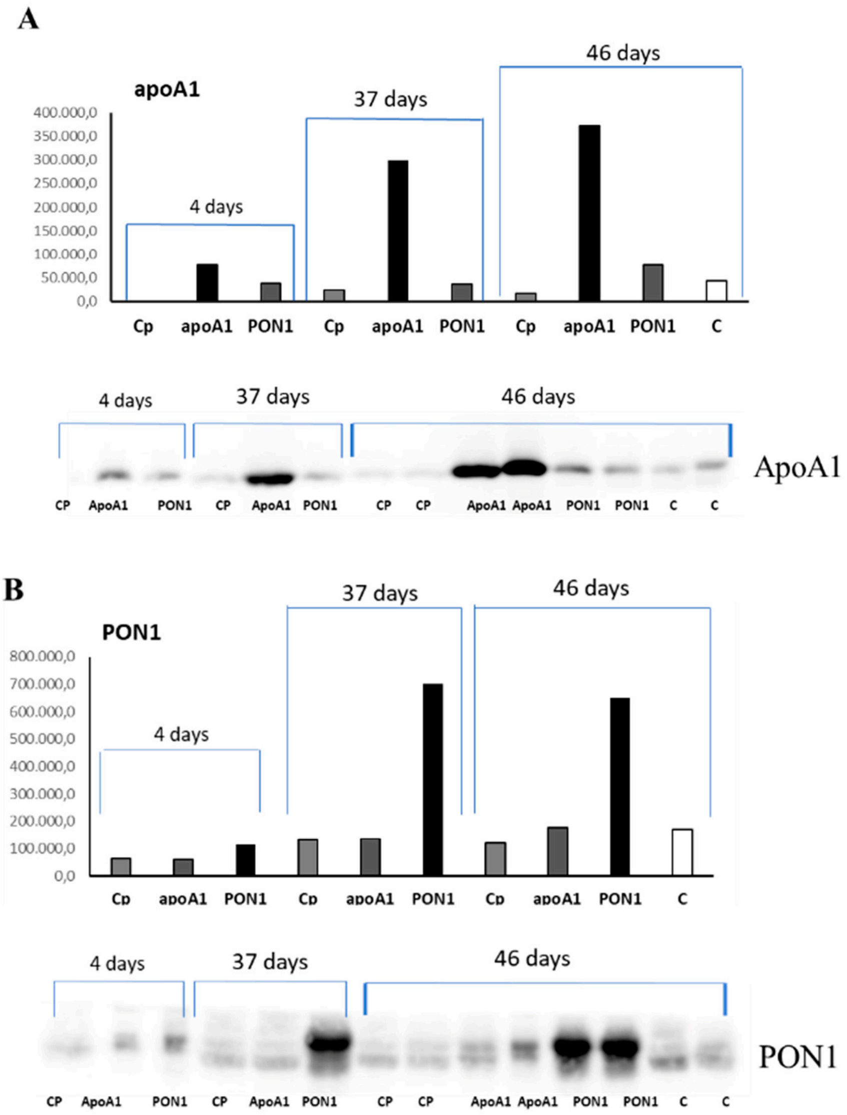

Figure S1. ApoAI and PON1 levels in the conditioned media from selected HuH-7, at different time points after CRISPR/dCas9 transfection. Densitometric analysis and representative Western Blot of ApoA1 (A) and PON1(B)

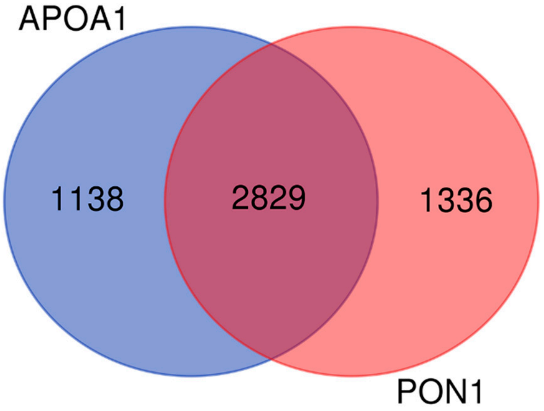

Figure S2. Venn diagram showing overlapping DEGs in APOA1 or PON1 transfection in hepatocytes.

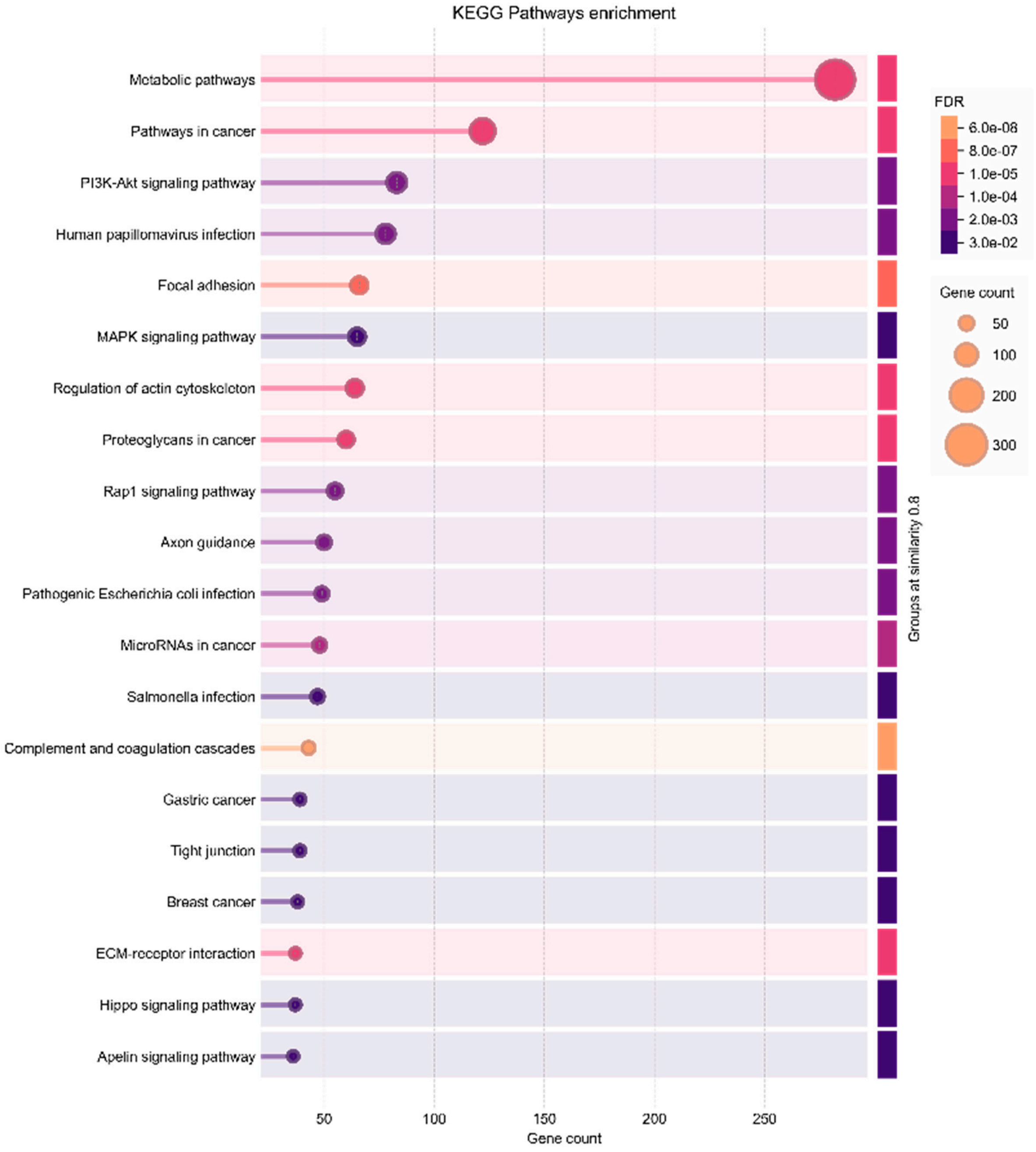

Figure S3. KEGG enrichment pathway analysis for shared DEGs after APOA1 or PON1 transfection in hepatocytes.
